# 基于GeoGebra的交互式色谱初学者学习工具开发与实践——以塔板理论为例

**DOI:** 10.3724/SP.J.1123.2025.03008

**Published:** 2025-09-08

**Authors:** Yuhan ZHANG, Junyao HE, Shujing LIN, Bingjian AI, Zhihong SHI, Hongyi ZHANG

**Affiliations:** 河北大学化学与材料科学学院，河北 保定 071002; College of Chemistry and Materials Science，Hebei University，Baoding 071002，China

**Keywords:** 仪器分析课程, 塔板理论, GeoGebra 平台, 交互式学习, 色谱流出曲线模拟, 数字化教学, instrumental analysis course, plate theory, GeoGebra platform, interactive learning, chromatographic elution profile simulation, digital teaching

## Abstract

针对仪器分析课程色谱教学中存在的理论表达抽象、知识传授单向、学生参与度低等问题，本文以塔板理论为切入点，构建了基于GeoGebra平台的交互式色谱学习工具。针对现有教学工具存在专业软件操作复杂、网页工具访问受限、参数调控自由度不足等缺陷，提出了“模型重构-工具开发-教学链融合”的创新型解决方案。通过整合流动相流速、死时间和相比等关键参数，建立改进型塔板理论数学模型；基于云端计算平台开发包含单组分模拟、多组分模拟和保留时间方程推导的三级进阶式学习模块；创新设计覆盖“数字化建模（‘豆包’AI辅助推导）-参数交互（多项可调色谱参数）-可视化验证（色谱流出曲线模拟）”的完整教学链。教学实践表明：（1）该工具突破了传统理论教学维度限制，课堂任务完成率提升至94%，学生高阶问题解答正确率提高至76%；（2）动态参数调控功能显著增强了学习参与度，85%的学生在后续学习中可自主使用该工具；（3）AI辅助推导与回归分析模块实现了理论化学与计算工具的跨学科融合，通过推导得出保留时间方程的做法比目前教材中直接给出结论的方式更具说服力。研究表明，这种“理论模型可视化-模型参数可调化-知识生成交互化”的创新模式，为解决色谱理论教学困境提供了新路径，其开源框架和模块化设计理念可为分析化学数字化教学改革提供有益参考。

## Abstract (en)

This study developed a GeoGebra platform-based interactive pedagogical tool focusing on plate theory to address challenges associated with abstract theory transmission， unidirectional knowledge delivery， and low student engagement in chromatography teaching in instrumental analysis courses. This study introduced an innovative methodology that encompasses theoretical model reconstruction， tool development， and teaching-chain integration that addresses the limitations of existing teaching tools， including the complex operation of professional software， restricted accessibility to web-based tools， and insufficient parameter-adjustment flexibility. An improved mathematical plate-theory model was established by incorporating mobile-phase flow rate， dead time， and phase ratio parameters. A three-tier progressive learning system （single-component simulation， multi-component simulation， and retention-time-equation derivation modules） was developed on a cloud-based computing platform. An integrated teaching chain that combined athematical modeling （AI-assisted “Doubao” derivation）， interactive-parameter adjustment （multiple adjustable chromatographic parameters）， and visual verification （chromatographic elution-curve simulation） was implemented. Teaching practice demonstrated that： （1） The developed tool transcends the dimensional limitations of traditional instruction， elevating the classroom task completion rate to 94% and improving the student accuracy rate for solving advanced problems to 76%. （2） The dynamic-parameter-adjustment feature significantly enhances learning engagement by enabling 85% of the students to independently use the tool in subsequent studies and experiments. （3） The AI-powered derivation and regression-analysis modules enable the interdisciplinary integration of theoretical chemistry and computational tools. The process of deriving chromatographic retention-time equations through this methodological approach proved more convincing than the current textbook practice of directly presenting conclusions. The developed innovative “theoretical-model visualizable-model-parameter adjustable-interactive-knowledge generating” model provides a new avenue for addressing teaching challenges associated with chromatography theory， and its open-source framework and modular design philosophy can offer valuable references for the digital teaching reform in analytical chemistry.

当前仪器分析课程教学存在着两个相互交织的困境^［1］^：其一，单向知识传输模式主导课堂，使学生陷入被动接受式的学习范式，严重制约了学习者主体性的发挥，导致高阶思维能力培养目标达成度偏低；其二，现代信息技术与课程体系的有机整合滞后，数字化教学资源开发不足。色谱分析是仪器分析课程的重要组成部分，其教学过程也存在上述问题。近年来，交互式模拟工具的出现为解决这些问题带来了新希望，能显著提升学生对抽象科学概念的理解^［2，3］^，但色谱法的交互式教学工具仍相对匮乏，尤其在兼顾理论深度与初学者友好性方面存在明显缺口。

已有研究报道了面向高阶学习者（包括专业化学从业人员）的色谱分离优化模拟软件^［4‒6］^，但这些软件因复杂性、操作专业性以及与学习者认知水平匹配度不足等原因，还难以适配本科生色谱分析入门课程的教学需求。Jordheim等^［7］^使用JavaScript编写代码开发了基于交互式网页的色谱教学工具，服务于塔板理论与基础色谱原理入门学习，但它发布在Github平台，不方便在我们国内课堂教学中使用。

在色谱分析的教学体系中，塔板理论常安排在教材和课堂讲授靠前位置。该理论由色谱技术的先驱Martin与Synge提出，已成为色谱界广泛接受的理论。尽管某些动力学模型（如速率理论）在理论上更为精准，但塔板理论仍然是色谱学教育的基石。GeoGebra作为一款优异的云端计算平台，在教学中发挥着越来越重要的作用。在总结了2010‒2020年有关GeoGebra教学应用期刊论文基础上，Yohannes等^［8］^得出这样的结论：利用GeoGebra的可视化功能可降低学习认知负荷。Kostic等^［9］^的工作证实，使用GeoGebra能有效提升学生解决溶液定量组成问题的学习成效。Lossjew等^［10］^将数字工具的基本教学功能与化学研究过程相结合，利用GeoGebra辅助探索了化学反应进程。冷却曲线及其数学模型为相变过程中的潜热、热传递、吉布斯相规则和质量传递动力学提供了丰富的信息。Shi等^［11］^通过将冷却曲线模型与GeoGebra绘图相结合的教学方法，加深了高年级本科生对复杂相变过程的理解，并有效增强了学生的数学建模、计算和解决问题的能力。我们^［12，13］^曾介绍过GeoGebra云端计算平台开展化学分析教学的应用案例。本文以塔板理论教学为切入点，以GeoGebra云端计算平台为教学环境，创建了相互衔接、渐次升级的3个学生活动模块（单组分色谱流出曲线模拟、多组分色谱流出曲线模拟和色谱保留时间方程），形成了“理论模型-问题案例-数字工具-可视化模拟”有机结合的教学链和学习链，提高了学生课堂教学中的参与度。本文所开发的塔板理论色谱图模拟过程具有如下突出优点：（1）工具开源和免费；（2）适用于手机、电脑等多终端，可在课堂教学中便捷运用；（3）可支持学生调节参数自主学习（如化合物数量、流动相流速、死时间、相比和塔板数等），增加学生课堂参与度和获得感。

## 1 理论模型的整合与重构

在大多数仪器分析课程的色谱教学体系中，教材内容和授课安排通常以塔板理论作为教学起点。教材中普遍会给出塔板理论数学模型，但较少将描述色谱过程的其他参数（如体积流速、死体积、死时间、相比和保留时间等）引入。本文将上述参数引入到塔板理论数学模型中建立了以流动相运行时间为自变量的综合模型，为构建交互式塔板理论教学工具奠定了化学基础。

### 1.1 塔板理论常见模型

若色谱柱有 n 块理论塔板，则色谱柱出口处流动相中的溶质浓度由式（1）给出^［7］^。式（1）是塔板理论的泊松函数，当塔板数 n 足够大时，非常接近于高斯分布函数。


$$C=\frac{1}{1+K}C_{0}\frac{1}{n!}e^{-v}v^{n}$$ （1）

式中， C 和 $C_{0}$ 分别是溶质在色谱柱出口的浓度及初始浓度， K 是分配系数。式（2）给出 v 的定义^［7］^，它表示与折合塔板体积有关的物理量，单位是“塔板体积”而非mL。


$$v=\frac{V}{Kv_{s}+v_{m}}$$ （2）

式中， V 是消耗的流动相体积， $v_{s}$ 和 $v_{m}$ 分别是每块塔板中固定相和流动相的体积。

### 1.2 塔板理论模型的近似变换

由于式（1）中存在阶乘的计算，运算量较大，不便于后期的模拟，因此要进行近似处理。当 n>10 ，阶乘运算可以按照Stirling关系式计算：


$$n!\approx \sqrt[{}]{2\pi n}{\left(\frac{n}{e}\right)}^{n}$$ （3）

将式（3）代入式（1），则有：


$$C=\frac{1}{1+K}C_{0}\frac{1}{\sqrt[{}]{2\pi n}}{\left(\frac{v}{n}\right)}^{n}e^{n-v}$$ （4）

### 1.3 塔板理论模型中时间变量的引入

经过上面的处理后，塔板理论模型的数学表达为式（4）所描述的动态方程及式（2）约束条件。若 K **、** $v_{s}$ 、 $v_{m}$ 、 n 和 $C_{0}$ 均为定值时，则 C 是 v 的函数，此时只能得到 C - v 图，而不是通常所见的 C - t 色谱图，因此需要将时间变量引入到式（2）约束条件中。

流动相的消耗体积 V 等于流动相的体积流速 F 与运行时间 t 的乘积：


V=F⋅t （5）

同理，死体积 $V_{m}$ 等于流动相的体积流速 F 与死时间 $t_{m}$ 的乘积：


$$V_{m}=F\cdot t_{m}$$ （6）

因为色谱柱由 n 块塔板构成，所以死体积等于每块塔板中流动相体积 $v_{m}$ 与 n 的乘积：


$$V_{m}=n\cdot v_{m}$$ （7）

相比 β 存在如下关系：


$$\beta =\frac{V_{m}}{V_{s}}=\frac{nv_{m}}{nv_{s}}=\frac{v_{m}}{v_{s}}$$ （8）

将式（5）~（8）代入到式（2），得：


$$v=\frac{V}{Kv_{s}+v_{m}}=\frac{Ft}{(K/\beta +1)v_{m}}=\frac{Ft}{(K/\beta +1)V_{m}/n}=\frac{tn}{(K/\beta +1)t_{m}}$$ （9）

至此，整合后的塔板理论模型数学表达包括式（4）所描述的动态方程及时间变量参数引入后的式（9）约束条件。这样当 K **、** $v_{s}$ 、 $v_{m}$ 、 n 和 $C_{0}$ 均为定值时， C 是 t 的函数，理论上可以得到符合常规色谱图形状的 C - t 图。

## 2 理论模型的教学应用

### 2.1 软件平台的选择

为了使学生在色谱课堂学习时可以获得自己模拟的色谱图，教学软件的选择是重要的环节。为了保证教学活动的顺利开展以及学生正常学习，需要选择一款开源免费软件平台。GeoGebra云端计算平台不仅开源免费，可以在手机、平板电脑和台式电脑等多终端运行，而且编程过程接近自然语言，非常适合在课堂环境下使用^［12，13］^。在此基础上，我们构建了一种塔板理论教学新模式。该模式依托相互衔接、渐次升级的3个学生活动模块（单组分色谱流出曲线模拟、多组分色谱流出曲线模拟和色谱保留时间方程推导），形成了“理论模型-问题案例-数字工具-可视化模拟”的教学链和学习链，提高了学生在塔板理论学习过程中的课堂参与度，使学生在理论认知深度、数字化建模能力、问题解决思维等方面同步提升。

### 2.2 学生活动模块1——单一组分色谱流出曲线模拟

为了使学生完整了解色谱分离流程，设计了色谱分离问题案例1：采用配备UV检测器的液相色谱仪分析组分A，色谱柱是 n=100 、 $V_{m}=3\;mL$ 和 β=2 的C18柱，流动相为水-甲醇混合液（60∶40， v/v），设定流速 F=1 mL/min 。已知组分A的初始浓度为1 mg/L，且它在流动相和固定相中的溶解度分别为 0.2 g/L 和0.6 g/L。请根据以上条件模拟出组分A的色谱流出曲线。

要利用式（4）和式（9）模拟色谱图，首先要指导学生结合题意和色谱基本理论，计算出死时间 $t_{m}$ 和分配系数 K 。其次，通过移动终端（手机/平板/笔记本）访问云端计算平台（https：//ggb123.cn/calculator）。表1为GeoGebra软件模拟色谱流出曲线的操作流程。参照表1提供的分步操作指南，在云端计算平台完成色谱流出曲线的参数化配置。执行完成这些流程后，系统将自动生成如图1所示的组分A色谱流出曲线。

**表1:** 用GeoGebra模拟单组分样品色谱流出曲线的流程

Line No.	Input	Comment
1	$C_{0}$ =1	initial concentration
2	$t_{m}=3$	dead time
3	β=2	phase ratio
4	n=100	number of plates
5	K=3	distribution coefficient
6	$v(t)=\frac{tn}{(K/\beta +1)t_{m}}$	Equation （9） ^*^
7	$f(t)=\frac{1}{1+K}C_{0}\frac{1}{\sqrt[{}]{2\pi n}}{\left(\frac{v(t)}{n}\right)}^{n}\cdot e^{n-v(t)}$	Equation （4） ^*^

* When typing equations （9） and （4） in GeoGebra， add parentheses after function symbols and specify the independent variable.

**图1:**
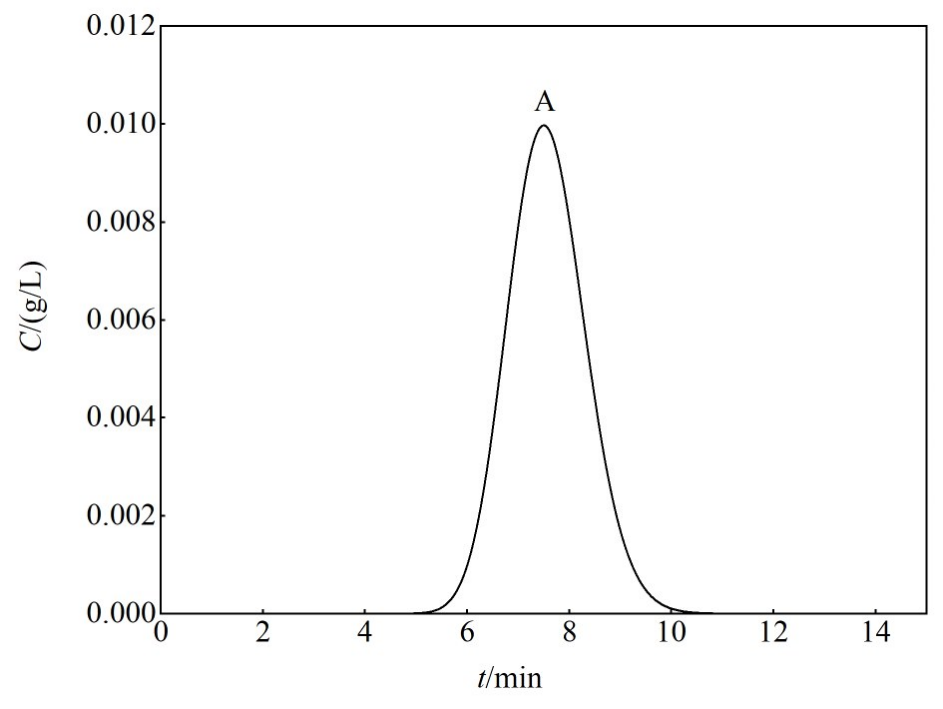
GeoGebra平台上单一组分的色谱流出曲线模拟图

本方法创新性地设计了一套动态参数调控机制，允许根据具体案例需求，灵活调整表1中第1至5行的核心参数（如初始浓度、死时间、相比、理论塔板数及分配系数等）。这一机制不仅确保了色谱流出曲线与问题背景的高度适配性，而且有效提升了学生的课堂互动参与度和学习获得感，增强学生解决问题的自信心。

### 2.3 学生活动模块2——多组分色谱流出曲线模拟

色谱分离案例2：在案例1的基础上，样品中增加另一组分B，其分配系数为1.8，问：组分A和B可以分开吗？

表2为用GeoGebra模拟分配系数 K=3和1.8 的两组分色谱流出曲线的操作流程。比较表2和表1会发现一些操作中的差异，其最大的不同在于输入式（9）和式（4）的变量个数有所不同，表1中这两个关系式均为一元函数，只是变量 t 的函数；而表2中这两个关系式均为二元函数，都是变量 t 和 K 的函数。图2为GeoGebra给出的色谱流出曲线模拟图。启发学生注意观察图2并与分配系数大小相联系，得到如下猜测：分配系数 K 大小会影响保留时间 t 长短，具体来讲就是 K 越大， t 越大； K 越小， t 越小。由案例1的单一组分色谱流出曲线模拟跨越到案例2的两组分色谱流出曲线模拟，让学生在亲自实操的基础上，对GeoGebra云端计算平台的操作技术以及对式（4）和式（9）模型的认知，都上升了一个层次。通过引导学生观察模拟色谱图，提升学生的观察力。

**表2:** 用GeoGebra模拟双组分样品（*K*=3和1.8）色谱流出曲线的流程

Line No.	Input	Comment
1	$C_{0}$ = 1	initial concentration of component A
2	$t_{m}=3$	dead time
3	β=2	phase ratio
4	n=100	number of plates
5	$v(t,K)=\frac{tn}{t_{m}(1+K/\beta )}$	Equation （9） ^*^
6	$f(t,K)=\frac{1}{1+K}C_{0}\frac{1}{\sqrt[{}]{2\pi n}}{\left(\frac{v(t,K)}{n}\right)}^{n}\cdot e^{n-v(t,K)}$	Equation （4） ^*^
7	f(t,3)	simulating profile of K=3
8	f(t,1.8)	simulating profile of K=1.8

* When typing equations （9） and （4） in GeoGebra， add parentheses after function symbols and specify the independent variable.

**图2:**
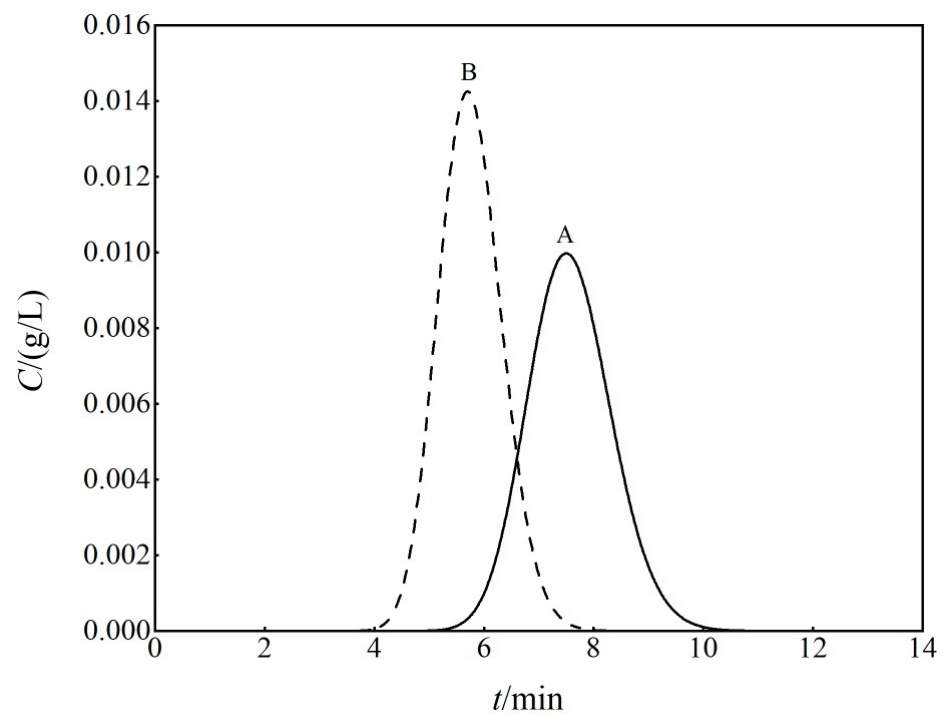
GeoGebra平台上两组分的色谱流出曲线模拟图

为了进一步验证从案例2产生的猜测，准备了案例3：在前述案例1的基础上，将样品中的组分数由1种增加到5种，分配系数*K*分别为1、3、5、7和9。

表3是处理案例3的操作流程。通过比较表3和表2的内容，可以看出它们的处理方法非常相似，表明模拟操作具有很强的延展性，可进行单组分和多组分的模拟。图3是按照表3操作流程由GeoGebra软件得到的5种混合物的色谱流出曲线。从图3中至少可以挖掘出两个有用的信息：其一，再次印证了前面的猜测—— K 值越大则保留时间越长；其二，在GeoGebra云端计算平台上，用手指点触色谱峰顶，即可方便地得到所对应的保留时间。于是可获得5组关于分配系数和保留时间的数据对，分别记为A=（1，4.5），B=（3，7.5），C=（5，10.5），D=（7，13.5）和E=（9，16.5），并标注在图3中。

**表3:** 用GeoGebra模拟不同分配系数（*K*=1，3，5，7，9）样品色谱流出曲线的流程

Line No.	Input	Comment
1	$C_{0}$ = 1	initial concentration of the sample
2	$t_{m}=3$	dead time
3	β=2	phase ratio
4	n=100	number of plates
5	$v(t,K)=\frac{tn}{t_{m}(1+K/\beta )}$	Equation （9） ^*^
6	$f(t,K)=\frac{1}{1+K}C_{0}\frac{1}{\sqrt[{}]{2\pi n}}{\left(\frac{v(t,K)}{n}\right)}^{n}\cdot e^{n-v(t,K)}$	Equation （4） ^*^
7	f(t,1)	simulating profile of K=1
8	f(t,3)	simulating profile of K=3
9	f(t,5)	simulating profile of K=5
10	f(t,7)	simulating profile of K=7
11	f(t,9)	simulating profile of K=9

* When typing equations （9） and （4） in GeoGebra， add parentheses after function symbols and specify the independent variable.

**图3:**
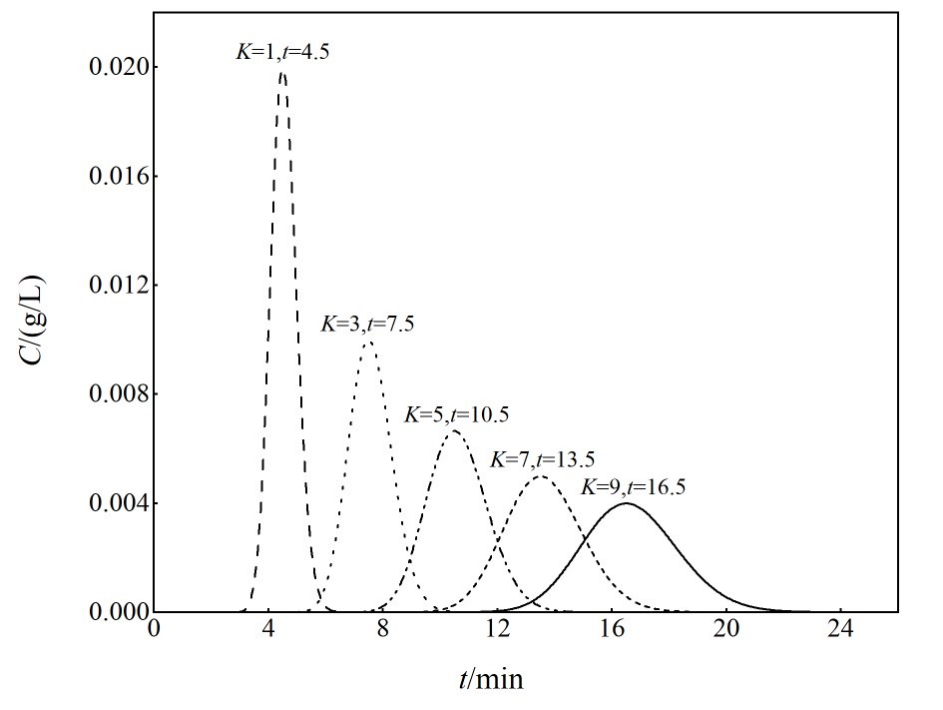
GeoGebra平台上5组分的色谱流出曲线模拟图

GeoGebra云端平台对仪器分析教学的另一个功能是可以直观地进行线性回归。表4为用GeoGebra进行保留时间与分配系数线性回归分析的详细步骤。从表4可见，利用GeoGebra进行线性回归操作非常直观，适合学生课堂参与探索。保留时间与分配系数存在着很好的线性关系，相关系数等于1，线性方程如下：


t=3+1.5K （10）

**表4:** 用GeoGebra进行保留时间和分配系数线性回归分析的步骤

Line No.	Input	Comment
1	A=（1，4.5）	K=1, t=4.5
2	B=（3，5，3）	K=3, t=7.5
3	C=（5，10.5）	K=5, t=10.5
4	D=（7，13.5）	K=7, t=13.5
5	E=（9，16.5）	K=9, t=16.5
6	fitline （｛A，B，C，D，E｝）	conducting linear fitting
7	corresponding coefficient （｛A，B，C，D，E｝）	showing correlation coefficient

式（10）使前面的猜想更加精准，表明保留时间与分配系数间存在着明确的线性依存关系，但线性关系的斜率和截距的化学意义是什么？以此问题引领学生深入思考，进入下一模块。

### 2.4 学生活动模块3——保留时间方程的推导

为了探究前述发现的保留时间和分配系数间的线性依存关系，解释式（10）中所蕴含的化学意义，引导学生重新审视前面的式（4）和式（9）色谱流出曲线模型。在所模拟的色谱流出曲线中最醒目的部分就是色谱峰，也就意味着式（4）中的浓度 C 存在极大值，就是说应有 $\frac{dC}{dt}=0$ 。为了避免学生陷入长时间数学推导过程而影响本课程的进度，决定采用国产AI模型“豆包（版本7.8.0）”（下文简称“豆包”）辅助推导。

首先，将式（9）和式（4）放到一起，如下：


$$v(t)=\frac{tn}{t_{m}(1+K/\beta )}$$ （9）

$$f(t)=\frac{1}{1+K}C_{0}\frac{1}{\sqrt[{}]{2\pi n}}{\left(\frac{v(t)}{n}\right)}^{n}\cdot e^{n-v(t)}$$ （4）

其次，将式（9）和式（4）拍成照片上传到“豆包”中，然后写一句“根据上图的两个式子，求 f(t) 导数等于零时的条件关系式”。“豆包”接到指令后就会给出以下3个详细步骤：分析 f(t) 的结构，利用乘积求导法则 ${\left(uv\right)}^{'}=u^{'}v+uv^{'}$ 求 f(t) 的导数，令 $f^{'}(t)=0$ 求解条件关系式，最终得到：


$$t=t_{m}(1+K/\beta )=t_{m}(1+k)$$ （11）

式中 k 代表着保留因子或分配比。式（11）就是常说的保留时间方程，它揭示了影响色谱保留时间的本质。当色谱柱选定和流速确定后，也就意味着相比 β 和死时间 $t_{m}$ 是不变的量，所以式（11）表明保留时间 t 就是分配系数 K 的函数，并从本质上证明了分配系数越大则保留时间越长这一前述实验观察和猜测的正确性。由式（11）可推知式（10）的初始实验条件为 $t_{m}=3$ 和 β=2 ，与表2所给初始条件一致。

目前教材中保留时间方程往往是直接给出的，逻辑上显得比较突兀，不利于学生创新思维的培养。本文从色谱流出曲线方程派生出保留时间方程这种做法，不仅拓宽了色谱流出曲线方程的应用范畴，锻炼了学生的逻辑思维能力和AI工具辅助学习的能力，而且增加了色谱课程的理论完整性。

## 3 教学实施和效果

数字化工具预学习（课前）：通过预习包（软件链接、操作指南及参考文献）引导学生熟悉GeoGebra云端工具。设置了创意作业：绘制“心”形函数 $x^{2}+{\left(y-\sqrt[{3}]{x^{2}}\right)}^{2}=1$ 图形并标注“我爱色谱课”。为了帮助学生顺利完成预习作业，提供了如下操作指南。第1步：在手机浏览器中键入ggb123.cn，进入图4a所示GeoGebra界面。点击该界面中的“启动计算器”，进入到工作界面（图4b）；可以把工作界面分为4个不同的功能区，从上到下依次为图形显示区（A区）、关系式键入区（B区）、键盘操作区（B与C之间的区域）、功能切换区（C区）。除此之外，右上角还有一个齿轮状按钮设置图标，可用于调整横纵坐标等操作，左上角的3条横线状的汉堡菜单，可用于保存文件等操作。第2步：点击C区的键盘操作区，即可在B区的关系式键入区录入： $x^{2}+{\left(y-\sqrt[{3}]{x^{2}}\right)}^{2}=1$ ，回车后则可在A区的图形显示区上观察到“心”形图案；然后，在C区下面的功能切换区中点击“工具”，从中选择“文本”，就可以录入“我爱色谱课”（如果没有切换成中文，可以直接键入英文）。学生通过“学习通”平台提交作业，预习作业提交率达100%，验证了趣味任务对学习动机的激发效果。

**图4:**
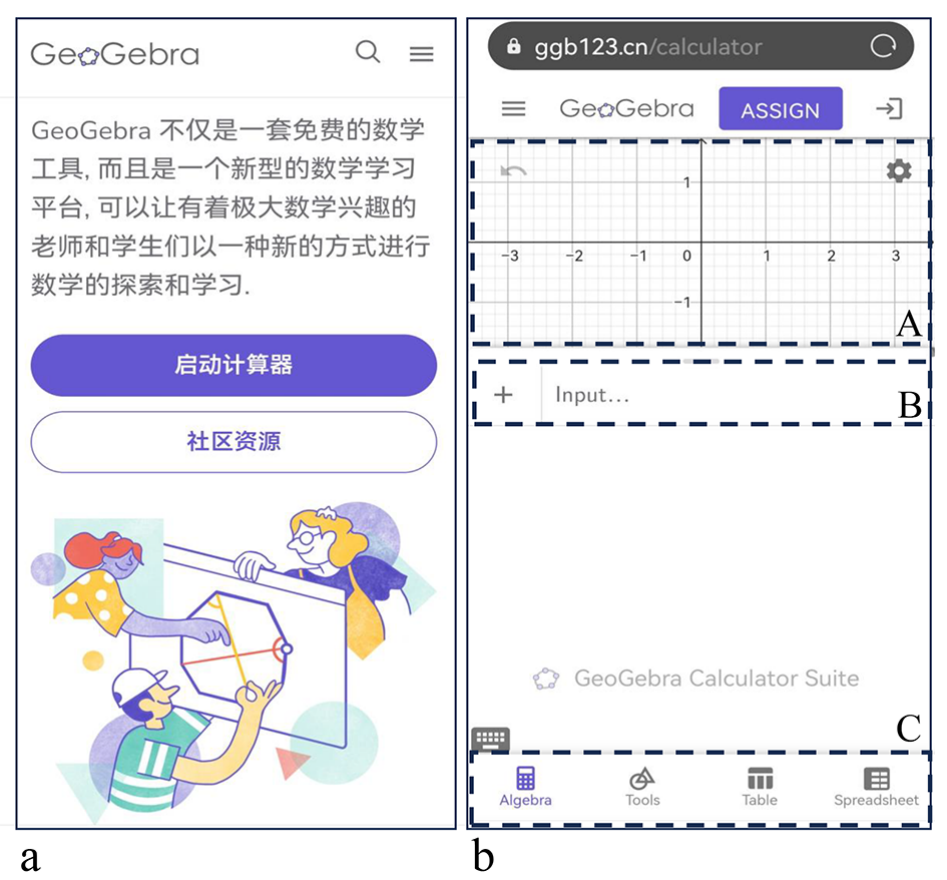
GeoGebra计算云平台的（a）界面和（b）工作界面

在课上模型建构阶段，我们采用知识迁移式教学。首先对比讲解塔板理论核心方程（泊松函数）与Stirling近似法的应用场景，采用“概念链”教学法，引导学生结合保留时间、死时间和相比等色谱参数，自主完成塔板理论模型约束条件的优化。我们重构了教材框架，构建了知识体系，并且通过知识迁移教学法使课堂互动频次提升了23%。在课上的模型应用阶段，采用三级进阶式模块教学。

模块1：单组分模拟。针对部分无色谱实验课程的专业（环境专业），为了丰富学生的色谱学习体验，案例1通过设计接近真实条件的色谱实验，提出模拟单一组分色谱流出曲线的任务。通过采用开源免费的云端计算平台并开展课前预热活动，学生在模拟单一组分色谱流出曲线时，实现了云端平台零障碍操作。实时生成模拟图谱，激发了学生自发展示学习成果的热情，94%的学生主动分享屏幕截图。

模块2：多组分模拟。在案例2模拟中，学生遇到的最棘手操作问题就是如何解决二元函数数值录入问题。教师通过及时微讲授的方式，重点突破学生遇到的二元函数输入技术难点。通过引导学生观察式（4）和（9），学生可以发现影响 C 的变量除了时间 t 以外，描述物质在固定相和流动相两相间分配的参数 K 也是重要因素，这样函数 C 和 v 就可看作 t 和 K 的二元函数了。在模块1中只把函数 C 和 v 当作 t 的一元函数，在直角坐标系中得到了 C - t 曲线的模拟图，这个经验对二元函数图的绘制有帮助，但还不能完全支撑直角坐标系中二元函数的模拟。二元函数图的模拟主要有两个关键步骤：第一步，键入标注二元变量的通式。按照表2和表3第5和6行那样操作，在键入式（4）和（9）通式时，需在函数符号 C 和 v 后的括号内注明二元自变量 t 和 K 。第二步，赋值并调用通式。按照表2第7行和第8行以及表3第7~11行那样操作，就可起到调用前述通式和将预设值赋值给自变量 K 的双重作用。回车后，GeoGebra平台接到上述操作指令就可在直角坐标系中完成二元函数的模拟。在实际操作中需要灵活运用一些操作技巧，比如通式中有两个符号含有字母 t 且其中一个还有下角标，为了提高键入速度且避免混淆，可以对表2中的字母含义作适当调整：可把表2中的 $t_{m}$ 均改为 t ，把原来的 t 都改为 x 。按照表2和以上提示内容操作后，就会看到图5的结果。图5中黑色方框内容就是上面提到的二元变量和自变量 K 的赋值。在大多数学生完成色谱流出曲线模拟后，我们组织学生开展针对色谱峰保留时间规律的小组讨论，引导学生形成有待验证的猜想。在案例3中，教师通过及时微讲授的方式，重点解决从色谱模拟图上读取保留时间的问题。当手指在手机屏幕上触碰色谱图峰顶时，屏幕上就可显示对应的横纵坐标数值。当学生完成系列保留时间-分配系数数据对采集后，教师通过实际操作演示，指导学生进行保留时间与分配系数之间的线性回归分析，最终得到对应的线性方程。线性方程中每一项蕴含着怎样的化学意义呢？借此引导学生展开深层次的思考。

**图5:**
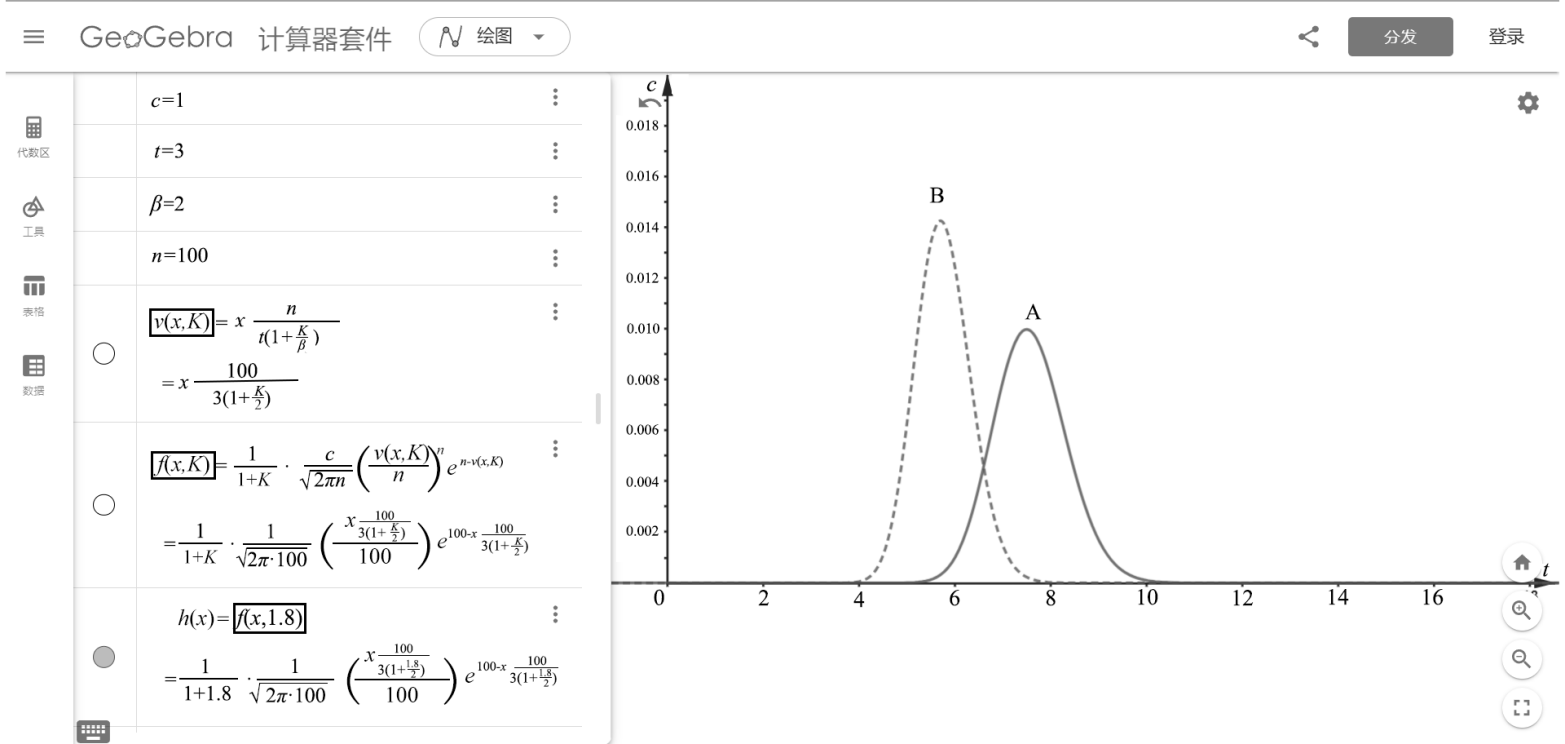
两组分色谱峰模拟的主要键入内容和流出曲线模拟结果界面

模块3：保留时间方程模块推导。我们首先将数学极值原理和色谱流出曲线的通式方程相结合，进而对塔板理论导数零点条件进行了推演。其次，开展AI助教辅助教学，运用“豆包”模型推演保留时间方程。最后，通过深入探究保留时间方程中分配系数与保留时间的内在关联，对前述线性方程的化学意义进行了阐释。

课后，结合学生的知识储备和学习能力，我们提供了一些涉及塔板理论推导的文献，供学生自主选择阅读。相较于传统授课模式，本文所介绍的方法借助“模型化-数字化-可视化”的流程，深度激发了学生在课堂上的参与积极性，显著提高了学生对色谱理论要点的掌握水平。例如，对于溶质在两相间的分配机制、理论塔板概念以及色谱峰生成机制等抽象知识，学生能够更好地理解和掌握。该教学模式具有以下两大优势：其一，可显著提升课堂教学效果。课堂任务完成率达94%，学生的高阶思维能力明显提高，保留时间方程推导正确率达76%。同时，89%的学生认为此教学模式在复杂理论的可视化解析教学方面成效显著。其二，运用GeoGebra解决色谱问题后，学生的长效迁移能力显著增强。例如，在后续的学习和实验中，85%的学生在速率理论最佳流速计算、色谱定量方法学习以及实验操作中，均能主动运用GeoGebra工具。这使得气相色谱程序升温和液相色谱梯度洗脱等高阶问题的解答正确率提升至76%。

## 4 结论

本研究通过整合GeoGebra平台与AI技术，成功开发了具有动态参数调节功能的三级进阶式色谱教学工具。该工具有效化解了塔板理论教学抽象性难题，使学生课堂任务完成率达94%，保留时间方程推导正确率达76%。实践表明，“数字化建模（‘豆包’AI辅助推导）-参数交互（多项可调色谱参数）-可视化验证（动态色谱流出曲线拟合）”模式可显著增强学生的参与度（85%的学生在实验中自主复用工具），其开源架构为分析化学数字化教学改革提供了可推广的实践范例。
